# Magnitude and determinants of suicide among overweight reproductive-age women, Chacha and Debre Berhan Town, Ethiopia: community based cross-sectional study

**DOI:** 10.1186/s13033-022-00551-w

**Published:** 2022-08-16

**Authors:** Abayneh Shewangzaw Engda, Habte Belete, Abate Dargie Wubetu, Nigus Alemnew Engidaw, Fetene Kasahun Amogne, Tebabere Moltot Kitaw, Tilahun Bete, Worku Misganaw Kebede, Bantalem Tilaye Atinafu, Solomon Moges Demeke

**Affiliations:** 1 Department of Psychiatry, College of Health Sciences and Medicine, Debre Berhan University, Debre Berhan, Ethiopia; 2 Department of Psychiatry, College of Health Sciences and Medicine, Bahir Dar University, Bahir Dar, Ethiopia; 3 Department of Midwifery, College of Health Sciences and Medicine, Debre Berhan University, Debre Berhan, Ethiopia; 4 Department of Psychiatry, College of Health Sciences and Medicine, Haramaya University, Harar, Ethiopia; 5 Department of Nursing, College of Health Sciences and Medicine, Debre Berhan University, Debre Berhan, Ethiopia; 6 Department of Psychiatry, College of Health Science Woldia University, Woldia, Ethiopia

**Keywords:** Suicidal ideation, Suicidal attempt, Overweight, Reproductive-age, Women

## Abstract

**Background:**

The magnitude and impact of women's suicidal behaviors, like suicidal ideation and suicidal attempts, are an important public health problem in low and middle-income countries, including Ethiopia. Suicidal behavior and being overweight are typical complications of reproductive age with many undesired consequences. Despite both having a serious impact on women of reproductive age, they are neglected in Ethiopia. Accordingly, this study aimed to examine the magnitude and determinants of suicide among overweight reproductive-age women in Chacha and Debre Berhan towns, Ethiopia.

**Methods:**

A community-based cross-sectional study design was once employed from April 1, 2020 to June 1, 2020. The Composite International Diagnostic Interview was used to measure suicidal attempts and ideation, and the data was collected by direct interview. All collected data were entered into Epi Data version 4.6 and analyzed with SPSS version 25. Bivariate and multivariable regression models were used to determine the factors associated with a suicidal attempt and ideation. A p-value of less than 0.05 was considered statistically significant.

**Result:**

Of the total participants, 523 were included, with a response rate of 93.7%. The prevalence of suicidal ideation was 13.0% (95% CI 10.1–15.9), whereas suicidal attempt was 2.3% (95% CI 1.1–3.6). Based on multivariable regression analysis, the odds of suicidal ideation have been higher among overweight women with stressful life events, depression, and younger age groups.

**Conclusion:**

Suicidal ideation was frequent in overweight reproductive-age women. Preventing, treating, and using coping mechanisms regarding identified factors is a good way to minimize the burden of suicide.

## Background

Suicide is an emergency mental health problem in the world that needs immediate action [1]. Even though the Sixty-third World Health Assembly adopted the principles of the World Health Organization (WHO) Mental Health Action Plan to reduce the rate of suicide by ten percent in different countries by 2020, the problem has accelerated over time [2].

Suicidal ideation and attempts are the preeminent contributors to the international burden of disease among women [3]. Besides the global burden, about three-fourths of it occurs in Ethiopia and other low and middle-income countries [4]. For instance, among an international study conducted in eleven “developing” and ten “developed” countries, the annual prevalence of suicidal behaviors has been marginally greater in developing countries [5]. However, little is understood related to the prevalence and modifiable associated factors of suicidal ideation and suicidal attempt [6, 7].

In traditional societies, suicide might be under-reported because of the huge level of stigma, religious and cultural condemnation. In Ethiopia, studies indicate that people who commit suicide are considered "denounced sinners" and can’t get the desirable funeral rituals but are buried in secluded areas. People who attempt suicide are "feared" and viewed as "cruel" and "inconceivable" [8, 9]

Gender-related vulnerability to psychopathology and psychosocial stressors is greater in women than in men in terms of morbidity and mortality from suicidal behaviors (suicidal ideation and attempt) [10].

Abnormal body weight causes serious mental health problems and affects the quality of life [11]. Overweight and suicidal ideation are the two most accelerated and important public health problems in the world [12, 13]. According to the Ethiopian Demographic Health Survey report, in urban Ethiopia, the proportion of overweight women was 12.1% [14].

The psychopathological aspects of suicidal ideation and attempts among overweight women are numerous and complex. Scholars discovered, however, that various factors, such as childhood sexual abuse, psychiatric disorders, non-partner physical violence, intimate partner violence, ever being divorced, widowed, or having a mother who had experienced intimate partner violence, had significant associations [3, 15, 16]. Having an appropriate weight status improves women's mental health by decreasing their negative emotions like shame, stigma, and negative self-concept associated with being overweight [17].

According to the WHO global mental health assessment carried out among females, the prevalence of 12-month suicidal attempts was 14.6% in developed countries and 19.2% in developing countries [5]. The prevalence of suicidal ideation in reproductive-age women in low-income countries ranges from 7.2% to 29.0% in Tanzania and Peru, respectively, whereas suicidal attempts in Tanzania were 0.8% up to 12.0% in Peru City [3]. Suicidal ideation was 16.5% [18] among overweight 40–49-year olds in Korea. Another study in Korea also revealed that suicidal ideation and attempts were 22% and 1.1%, respectively [19].

Different studies in Ethiopia revealed that the minimum lifetime prevalence of suicidal attempts was 0.8%, whereas the maximum prevalence limit was 14.8% [20, 21]. The annual prevalence of suicidal behavior was 7.9 in the general population, and suicidal attempts among females were 32.0% [22].

Even though suicide remains one of the most common causes of death, little attention is paid to it, especially for overweight women. Suicidal behaviors such as suicidal ideation and attempt and being overweight are typical complications of reproductive-age women with many undesired consequences. However, there has been little research into the extent to which overweight women in Ethiopia have experienced suicidal ideation and attempts, as well as the factors that contribute to these behaviors. Thus, this study aimed to examine the magnitude and determinants of suicide among overweight childbearing age women in Chacha and Debre Berhan towns, Ethiopia. This study will be used by the Chacha and Debre Berhan town health bureaus to call for the prevention of women's suicidal behaviors and other related health issues by increasing clinical awareness and using a cross-disciplinary care approach. Moreover, policymakers and planners will take it as input to find appropriate measures to overcome problems concerning suicidal ideation and attempts.

## Methods

### Study design and setting

The study was a community-based cross-sectional survey conducted among reproductive-age women in Chacha and Debre Berhan towns from April 1, 2020, to June 1, 2020. Chacha town is one of the towns in the Amhara region, North Shoa Zone, Angolela and Tera woreda. The town had 2641 reproductive-age women and only one health center and four private clinics. Whereas Debre Berhan is one of the towns in the Amhara region, which is located 130 kms to the north of Addis Ababa, the capital city of Ethiopia. The town had 23,093 reproductive-age women [23]. Only one governmental comprehensive specialized hospital, four health centers, and one private general hospital are found in the town. Moreover, psychiatric services were delivered only at the comprehensive specialized hospital at the outpatient level. Although in the two towns, the problem reported is that the community, via different sources and direct professional experience, and the population have a difference in living situations and infrastructure, they get mental health services only from Debre Berhan Comprehensive Specialized Hospital. So, in order to determine the distribution and magnitude of the problem in the two towns, and based on the research findings to broaden the service, we began to select the two sites.

#### Study participants

The source population was all childbearing age women at household level living in Chacha and Debre Berhan towns, whereas the study population was women who were overweight, resided for at least 6 months in the study area, and were available during the data collection period.

#### Inclusion criteria

The sample includes participants aged 15 to 49 years old who have resided for at least 6 months in the study area.

#### Exclusion criteria

Women in their second and third trimesters who are edematous, unable to communicate, or have spinal problems.

### Sample size determination and sampling technique

In this study, Epi Info version 7 was used to compute the sample size by bearing in mind the following assumptions: 95% confidence level, the margin of error of 5%, the expected prevalence of suicidal behaviors at 50%, the design effect of 1.5, and the total population size in both study areas of 2,790. Therefor, the overall sample size is N = 507. When we add 10% non-response, the working sample size is N = 558.

**Fig. 1:**
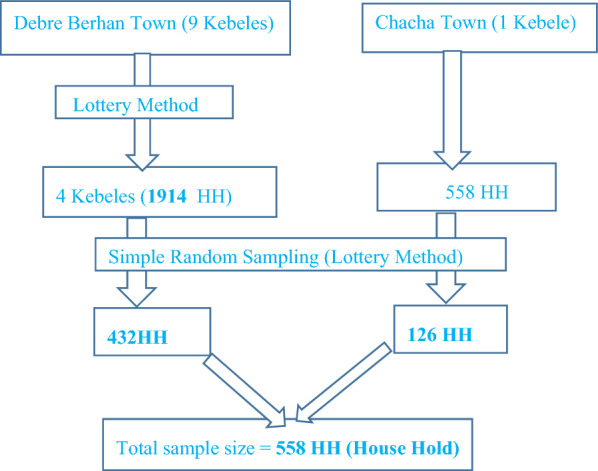
Sampling procedure of overweight reproductive age women in 2020

Chacha town has only one kebele (the smallest governmental administrative structure in Ethiopia). While Debre Berhan town has nine administrative kebeles in its study area, four kebeles were chosen at random.Then, in each kebele, a preliminary assessment was performed to get the number of households that have overweight women. Based on the survey findings, 632 overweight reproductive-age women within 558 households and 2158 overweight reproductive-age women within 1914 households were found in Chacha and Debre Berhan towns, respectively. The list of household numbers from a survey database was used as a sampling frame. Based on the proportionate allocation formula, from Chacha town 126 and Debreberhan 432 participants were recruited by using a simple random sampling technique. The lottery method was used to identify the participants in each selected Kebeles (Fig. 1).

### Data collection procedures

A total of 10 female diploma nurses for data collectors and two BSc psychiatric nurses for supervisors were recruited for data collection. Training was given for two days on the objectives of the study; assessment tools; data collection approach; privacy; and confidentiality. A face-to-face interview using a semi-structured questionnaire (unstructured questionnaire like socio-demographic data, chronic illness, stillbirth, abortion, history of mental illness, and a structured questionnaire including suicidal ideation and attempt, depression, substance use, intimate partner violence, social support, stressful life events, and weight stigma experienced) was employed to collect the data, which was prepared in the local Amharic language.

### Measurements

#### Suicidal ideation and attempt

A locally validated Amharic version of the World Health Organization (WHO) composite international diagnostic interview (CIDI) tool was used to calculate suicidal ideation and attempt. This tool uses the suicidality module of the World Mental Health (WMH) survey initiative version [24, 25].

#### Depression

It was measured by using a nine-item patient health questionnaire. Depression has been considered if the participants' nine-item patient health questionnaire score was ≥ 5 and the tool was validated in East Africa, including Ethiopia. For an institutional setting, the cutoff point is 10, with a sensitivity of 86% and specificity of 67% [26].

#### Body mass index (BMI)

BMI was measured by dividing the weight in kilograms by the height in meters squared. Overweight was declared with a range of BMI values from 25 kg/m^2^ to 29.9 kg/m^2^ [27].

#### Experienced Weight Stigma

A ten-item modified version of the Stigmatizing Situations Inventory (SSI) was employed to measure the experience of weight stigma within the past month. It has a six-point Likert scale ranging from 0 (never) to 5 (daily). The internal consistency was = 0.92, indicating that the data was sufficiently reliable [28, 29].

#### Body image disturbance (BID)

A single item (“I feel satisfied with the shape of my body”) is used to verify BID. The response score ranged from zero (always) to five (never) and if the women scored above the mean end result, it was considered to reflect a higher degree of BID [30].

#### Stressful life events

A List threatening experience questionnaire was employed to measure stressful life events with a score of zero to six. Scores ≥ one take it as having to experience stressful life events. The tool has good test–retest reliability, and the inter-rater reliability revealed Kappa values of 0.61 to 0.87 [31, 32].

#### Social support

The three-item Oslo social support scale was used to identify social support status. The scores indicated that 3–8 = poor social support, 9–11 = moderate social support, and 12–14 = strong social support [33].

#### Intimate partner violence

Eight-item Woman Abuse Screening Tool (WAST) was employed to assess intimate partner violence. WAST assessed physical, sexual, and emotional abuse in the last 12 months, and the participants were considered exposed to intimate partner violence when the score ≥ 4 symptoms. It received a score of zero (never), one (sometimes), and two (often). It has 91.7% and 100% specificity and sensitivity, respectively [34, 35].

#### Substance use history

It was screened by the Alcohol, Smoking, and Substance Involvement Screening Tool (ASSIST) [36].

### Data processing and analysis

After checking all questionnaires for completeness, the data was coded appropriately and entered into a computer using Epi-data version 4.6 statistical programs. Then, the data set was transferred to SPSS version 25 for analysis. Frequency distribution and percentage were used to summarize the data, and text and tables were used to present the data concerning socio-demographic and other relevant variables. A bivariate analysis was used to see the association between suicidal ideation and attempt and predictor variables. Predictor variables with a P-value of less than 0.25 were selected as candidates for multivariable analysis to control the effect of confounding variables. Finally, variables that had a significant association with the outcome variable were determined based on AOR with 95% CI and p-value < 0.05.

## Result

### Socio-demographic characteristics of participants

From 558 participants, 93.7% had reacted to the complete information. The median age of participants was 31 years, with a range of 15–49 years. The majority (40.0%) of the ages ranged from 15–24 years. One-third of participants were housewives, 152 (29.1%), followed by around one-fourth were government-employed, 138 (26.4%). Nearly 90% of participants were Orthodox Tewahdo Christian followers and the Amhara (96.9%) ethnicity. Regarding family average monthly income, 451 (86.20%) were above the poverty line (Table 1).

**Table 1:** Socio-demographic characteristics of overweight reproductive-age women in Chacha and Debre Berhan town 2020

Variables	Overweight women N = 523	
	Frequency	Percent
Age		
15–24	102	19.5
25–34	212	40.5
35–44	158	30.2
45–49	51	9.8
Religion		
Orthodox Tewahdo	465	88.9
Protestant	43	8.2
Muslim	12	2.3
Others*	3	0.6
Ethnicity		
Amhara	507	96.9
Oromo	11	2.1
Others**	5	1.0
Marital status		
Married	299	57.2
Single	181	34.6
Divorced	33	6.3
Widowed	10	1.9
Educational status		
No formal education	23	4.4
Primary school	61	11.6
Secondary school	138	26.4
College/university	301	57.6
Family monthly income		
Above the poverty line	451	86.0
Below poverty line	72	14.0

Others*: Yehiwa miskir, Catholic and Mulu wengel; **Tigrie & Debub nation & nationalities

### Substance use history, clinical and psychosocial factors-related characteristics of reproductive-age women

Out of the total participants, 414 (79.2%) and 7 (1.3%) used alcohol and khat in their lifetime, respectively, but there is no report of smoking. Only 3 (0.6%) participants had alcohol use disorders, while 7 (1.3%) had khat use disorders. Additionally, 49 (9.4%) participants had an abortion, whereas 19 (3.6%) experienced stillbirth.

Twenty-seven (5.2%) participants had chronic medical illnesses and hypertension was frequently reported at 16 (3.1%), followed by diabetes mellitus at 7 (1.3%) and others (HIV/AIDS, epilepsy) at 4 (0.8%). Of the overall participants, only two overweight women had a history of mental illness without incorporating depression. On the other hand, depression among the family was not reported. Beyond that, half of the participants had moderate social support 271 (51.8%). Additionally, out of the total participants, one-sixth had stressful life events, one-third had depressive symptoms, 214 (40.9%) experienced stigma, and 232 (44.4%) had body image disturbance (Table 2).

**Table 2:** Substance and psycho-social factors-related characteristics of study participants among overweight reproductive age women in Chacha and Debre Berhan town 2020

Variables	Category	Overweight women N = 523	
		Frequency	Percent
Ever alcohol use	Yes	414	79.2
	No	109	20.8
Ever khat use	Yes	7	1.3
	No	516	98.7
Depressive symptoms	Yes	172	33.0
	No	351	67.0
Body image disturbance	Yes	232	44.4
	No	291	55.6
Social support	Poor	144	27.5
	Moderate	271	51.8
	Strong	108	20.7
Stressful life events	Yes	78	14.9
	No	445	85.1
Experienced weight stigma	Yes	214	40.9
	No	309	59.1

### Characteristics of suicidal behaviors (suicidal attempt and ideation)

The total prevalence of suicidal ideation and suicidal attempts among overweight child-bearing age women was 13.0% (95% CI 10.1–15.9) and 2.3% (95% CI 1.1–3.6), respectively. In the case of the suicidal plan, the prevalence of suicidal plan was 3.3% (95% CI 1.9–5).

Regarding the frequency of lifetime suicidal attempts, 10 (83.3%) attempted suicide once and 2 (16.7%) attempted suicide twice.

Three-fourths of suicidal attempt participants used poison, and two respondents by hanging and one by using medication overdose. This was due to family conflict 2 (17.0%), poverty 3 (25.0%), mental illness 1 (8.0%), and physical illness 6 (50.0%). All respondents who attempted suicide attempted to kill themselves with the real intent of dying.

### Associated factors of suicidal behaviors among overweight reproductive-age women

Variables in the bivariate analysis with a p-value less than 0.25 were selected as a candidates for multivariable analysis and the significance level was declared with a p-value < 0.05.

Accordingly, the odds of suicidal ideation among women with stressful life events were 2.30 times more likely than women who had not stressful life events (AOR = 2.30, 95% CI 1.20, 4.30). Suicidal ideation among women with a history of depressive symptoms was 11.60 times more likely as compared to those without a history of depressive symptoms (AOR = 11.60, 95% CI 5.90, 22.60).

Women with a young age group were 2.80 times more likely to experience suicidal ideation than those whose age group was 35 to 44 (AOR = 2.80, 95% CI 1.23, 6.22), but body image disturbance, abortion, experienced bodyweight stigma, and marital status were not significantly associated (Table 3).

**Table 3:** Bivariate and multivariable analysis associated factors of suicidal behaviors among overweight reproductive-age women in Chacha and Debre Berhan town, 2020

Variables	Response	Suicidal ideation		COR (95%, CI)	AOR (95%, CI)
		Yes	No		
Age	15–24	19	83	1.67 (0.84, 3.34)	2.80(1.23, 6.22)**
	25–34	26	186	1.00 (0.54, 1.90)	1.44(0.70, 2.90)***
	45–49	4	47	0.60 (0.20, 1.90)	0.80(0.25, 2.90)***
	35–44	19	139	1	1
Stressful life events	Yes	25	53	4.40 (2.50, 7.80)	2.30 (1.20, 4.30)**
	No	43	402	1	1
Depressive symptoms	Yes	55	117	12.20 (6.45, 23)	11.60 (5.90, 22.60)*
	No	13	338	1	1
Ever alcohol use	Yes	58	356	1.60 (0.80, 3.30)	2.00 (0.90, 4.43)***
	No	10	99	1	1

*p-value ≤ 0.0001 (highly significant), **p-value ≤ 0.05(significant), ***p-value > 0.05, 1: Reference; COR: Crude odd ratio; AOD: Adjusted odd ratio

## Discussion

This study revealed that the prevalence of suicidal ideation and suicidal attempts among overweight childbearing age women was 13.0% and 2.3%, respectively. Stressful life events, depressive symptoms, and being younger were discovered to be associated factors.

Based on this study, the prevalence of suicidal ideation is higher as compared to studies done in Australia (4.5%) [37], and China (4.9%) [38]. The discrepancy might be due to the difference in study design, the time of data collection, cultural differences, participant age variation, and environmental conditions. It is lower when compared to the study done in a different area of Korea (29.8%) [18], (22%) [19], (18.3%) [39], USA (21.6%) [40] and the United States (22.1%) [41]. The difference could be due to cultural differences, participant age differences (18 years among Korean women versus 14 years in the United States), and also in some Korean studies that include the general overweight population, whereas this study only included overweight reproductive-age women.

In the case of suicidal attempts, the prevalence is lower in this study when compared to studies in Korea [42], (3.6%) [39], USA (8.7%) [40] and the United States (11.0%) [41]. Normative attitudes towards suicide across diverse cultures, religions, and economic status might be part of the reason for the discrepancy. For instance, studies in Ethiopia showed that suicide was considered “condemned sinners” and people who had suicidal attempts were “feared” and labeled “cruel” and “untrustworthy” [8]. However, suicidal behavior has not yet been studied among overweight reproductive-age women in Ethiopia.

Regarding the associated factors of suicidal ideation, being an adolescent and within the age group of 18 to 24 is 2.80 times more likely than the age group of 35 to 44, and this result is encouraged by a study done in Korea [19, 42]. The possible reasons might be due to rapid hormonal change, increased use of the substance, and social engagement complexity.

Concerning depressive symptoms, they were 11.60 times more likely to develop suicidal ideation than those who had no depressive symptoms. This result was supported by a study conducted in Korea [18, 43]. This might be due to low levels of brain-derived neurotrophic factor [44] and depression leading to hopelessness; it is a robust predictor of suicide [45].

A stressful life event is one of the significant etiological factors for suicidal ideation. Overweight women who experienced stressful life events were 2.30 times more likely to experience suicidal ideation than those who had not. This outcome was consistent with a systematic review meta-analysis in the USA [46–48]. Stress results in a long-lasting change in the brain morphology and biology, and this changes the functional states of various neurotransmitters, 5-hydroxytryptamine (serotonin) 2A receptor gene polymorphisms [49] and intraneuronal signal system, changes that loss of neurons and excessive reduction in synaptic contact, and also stress decreases numerous brain proteins that are the baseline for neuronal growth and synapse formation. This leads to suicidal ideation [49, 50].

## Strength of the study

The present study was done with a large sample size at the community level and in two towns using a validated tool and good sampling techniques. Since there is no previous study done on suicidal ideation and suicidal attempts among overweight women in Ethiopia, this research will serve as a getaway for other studies among different societies that have different religious and other cultural values.

## Limitations of the study

Due to social desirability bias, suicidal behaviors may be miscalculated. The study did not incorporate underweight, normal weight, and obese women. Furthermore, the study design does not allow an accurate assessment of the causal association between overweight and suicidal risk.

## Conclusion

This study confirms that suicidal behaviors (suicidal ideation and attempts) against overweight reproductive-age women who reside in Chacha and Debre Berhan are commonly observed. Suicidal risk assessment and prevention should be given special attention to vulnerable individuals in order to address multiple factors such as depressive symptoms, stressful life events, and the young age group. A further cohort study is indicated to determine the cause and effect relationship between specific risk factors for suicidal ideation and attempt.

## Data Availability

The corresponding author can bring the datasets collected and analyzed for this study based on a reasonable request.

## Abbreviations

BMI: Body Mass Index
BSc: Bachelor of Science
CI: Confidence Interval
CIDI: Composite International Diagnostic Interview
PHQ-9: Patient Health Questionnaire
SPSS: Statistical Package for Social Science
SSI: Stigmatizing Situation Inventory
WHO: World Health Organization
USA: United State of America
